# From Underutilized Tuber to High-Value Food Resource: Advances in Bioactive Components, Processing Technologies and Sustainable Valorization of Tiger Nut (*Cyperus esculentus* L.)

**DOI:** 10.3390/foods15213872

**Published:** 2026-10-29

**Authors:** Li Li, Tianle Yao, Xiaoke Zhang, Qiuyi Du, Mengyuan Zhang, Qian Zhang, Mengtian Huang, Liling Wang, Yu Han, Long Wu

**Affiliations:** 1 Hubei Key Laboratory of Resource Utilization and Quality Control of Characteristic Crops, College of Life Science and Technology, Hubei Engineering University, Xiaogan 432000, China; 2 National “111” Center for Cellular Regulation and Molecular Pharmaceutics, Hubei University of Technology, Wuhan 430068, China; 3 College of Food Science and Engineering, Tarim University, Alar 843300, China; 4 Key Laboratory of Tropical Fruits and Vegetables Quality and Safety for State Market Regulation, School of Food Science and Technology, Hainan University, Haikou 570228, China

**Keywords:** tiger nut (*Cyperus esculentus* L.), value-chain upgrading, multi-omics, bioactive components, processing modification, functional food, by-product recycling, cascading biorefinery

## Abstract

Tiger nut (*Cyperus esculentus* L.) is a perennial herb of the family Cyperaceae whose underground tubers supply oil, starch, protein and dietary fiber, making it an emerging composite tuber resource. Earlier reviews described composition or addressed oil alone; here, structure–function relationships are the analytical core, and this review asks how molecular and structural knowledge of its components can be translated into designed functionality within a value-chain upgrading frame. A systematic search of four databases yielded 100 peer-reviewed studies together with the four standards and regulations that govern the sector, which were assessed on a common evidence scale separating mechanistic findings from correlative ones. Multi-omics has identified the genes and networks underlying tuber enlargement and the high-oil, high-oleic-acid phenotype. Tuber lipids, polyphenols, peptides, polysaccharides and dietary fiber display antioxidant, gut microbiota-modulating, metabolic and immunomodulatory activities; however, human clinical evidence remains scarce. Green extraction, physical-field and enzymatic treatments can direct the structural control and functional enhancement of oil, protein and starch, supporting plant-based beverages, bakery products, fat replacers and Pickering delivery systems, while cascading biorefinery converts processing residues. Transfer from laboratory prototype to industrial product is blocked by unresolved structure–activity relationships, weak evidence grading, imprecise processing and absent standardization; research and industrialization priorities are proposed.

## 1. Introduction

**Figure 1:**
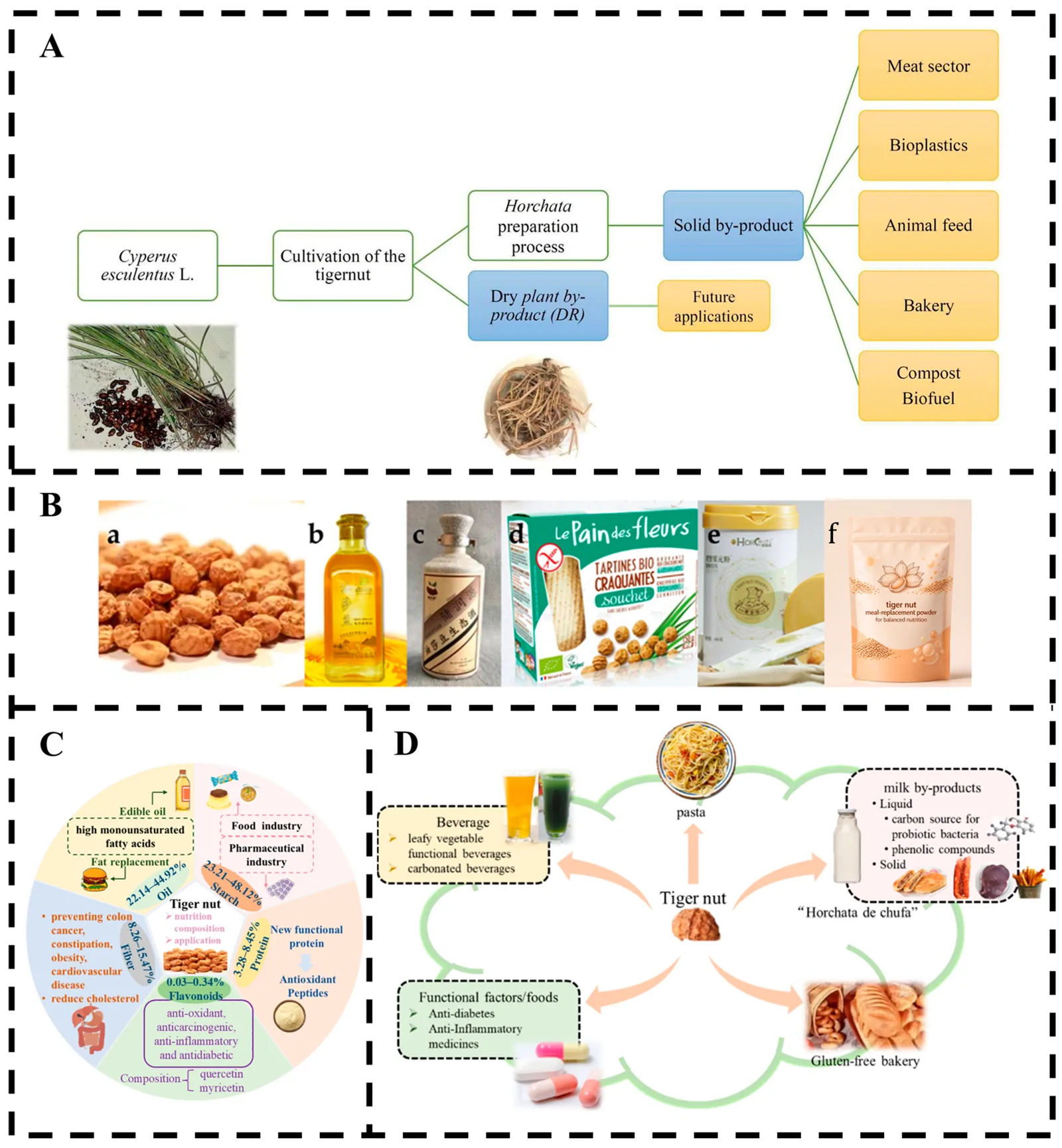
From tuber to multi-product value chain: Compositional basis and food applications of tiger nut (*Cyperus esculentus* L.). (**A**) Side streams generated during the preparation of tiger nut milk (horchata), illustrating that processing yields several distinct by-products rather than a single residue [6]. (**B**) The tuber and representative commercial products: (**a**) Tuber, (**b**) edible oil, (**c**) liquor, (**d**) crunchy bio toast, (**e**) solid powder drink and (**f**) meal replacement powder [4]. (**C**) Major nutritional constituents of the tuber and the nutrient-based applications they support [4]. (**D**) Applications of the whole tuber in the food industry [4]. Reused panels are licensed under CC BY 4.0.

Tiger nut (*Cyperus esculentus* L.) originated along the Mediterranean coast and in Africa and is now widely distributed in temperate and tropical regions. As a tuber crop that simultaneously supplies oil, starch and protein, its tubers contain up to 20–36% oil on a dry-weight basis, dominated by oleic acid with a high proportion of monounsaturated fatty acids [1], and are also rich in soluble dietary fiber, resistant starch, polyphenols and minerals, giving them a nut-like nutritional structure and functional potential [2]. In Europe, the traditional tiger nut beverage ‘horchata de chufa’ has been consumed for several centuries, and its raw material ‘Chufa de Valencia’ is protected under the European Union Protected Designation of Origin (PDO) scheme [3]; in Africa and West Asia, tiger nut remains an important traditional food and oil source [4]. With the rise of plant-based foods, functional foods and green processing, tiger nut is moving from a regional traditional food toward a global high-value food ingredient, drawing on its combined advantages of high oil content, high fiber content and sustainable cultivation [5]. This composite composition determines the range of product formats and the pattern of by-products that originate from the tuber (Figure 1).

This transition, however, is not merely a change in market positioning; it rests on a set of five open scientific questions. (i) Why can tiger nut tubers accumulate oil and starch so efficiently? (ii) Which key genes and regulatory networks determine the high-oil and high-oleic-acid phenotype? (iii) At what level of evidence do the health functions of its bioactive components actually stand? (iv) How can processing technologies achieve directed structural modification and functional enhancement of these components? (v) And how can processing residues be brought into a recycling system that supports the sustainability of the industry? Answering these questions requires moving beyond the conventional three-part review scheme of ‘composition—bioactivity—utilization’ and reintegrating the field from the systemic perspective of ‘resource—components—function—processing—product—recycling’. These five questions are addressed in sequence in the following sections. The mechanism of oil and starch accumulation and the genes and regulatory networks that determine the high-oil, high-oleic-acid phenotype are treated in Section 2; the level of evidence supporting the health functions of the bioactive components is graded in Section 3; the processing strategies available for directed structural modification and functional enhancement are examined in Section 4; and the routes by which processing residues can be brought into a recycling system are considered in Section 6.

A transparent literature search was carried out in Web of Science Core Collection, Scopus, PubMed and Google Scholar. The core terms ‘*Cyperus esculentus*’, ‘tiger nut’, ‘chufa’ and ‘yellow nutsedge’ were combined with the Boolean operators AND/OR with the descriptors ‘oil’, ‘protein’, ‘starch’, ‘dietary fiber’, ‘polyphenol’, ‘bioactive peptide’, ‘processing’ and ‘residue’, and the search was restricted to English-language peer-reviewed original research articles and reviews; no lower date limit was applied, and the final search was run in October 2026. Records were screened first by title and abstract and then by full text; studies dealing exclusively with weed control and unrelated to food science, records without accessible full text, conference abstracts and duplicated publications were excluded. The search returned 1045 records in total; after removal of 187 duplicates, 662 records were excluded at the title and abstract stage, and 196 records were assessed in full text. A total of 100 peer-reviewed studies together with four standards and regulations met the eligibility criteria and are cited in this review, and the screening decisions were logged to keep the process traceable and reproducible.

Drawing on this systematic synthesis, this review adopts value-chain upgrading as its organizing thread. Starting from the resource biology of tiger nut, it examines, in turn, the bioactive components and health functions, processing extraction and directed functionalization, high-value food applications and by-product recycling, and closes with the remaining challenges and future directions. Compared with existing reviews [1,5], the contribution of this review lies in four respects, namely (1) placing fundamental composition research and food-processing application within a single logical chain that links ‘molecular mechanism’ to ‘product design’; (2) assessing health-function findings from the perspective of evidence grading, so as to avoid over-interpretation; (3) systematically summarizing processing strategies with emphasis on structure–function relationships; and (4) explicitly proposing a cascading biorefinery route for residues together with a pathway for industrial chain upgrading. Taken together, these four points define the specific contribution of this review relative to previous syntheses: it does not add another description of composition; instead, it assesses, on a common evidence scale, which structure–function relationships are mechanistically established and which remain correlative.

To position tiger nut explicitly against other crops, Section 2.2 sets its oil content and oleic acid share against those of the major edible oil crops and its protein and dietary fiber contents against those of the main starchy root and tuber crops, and reads that comparison as a strengths, weaknesses, opportunities and threats (SWOT) assessment; in essence, tiger nut is unusual in combining an oil content comparable to that of an oilseed [7] with the starch-rich storage habit of a root or tuber crop [8].

## 2. Resource Biology and Nutritional Composition of Tiger Nut

Understanding why tiger nut can become a high-value tuber is a prerequisite for its high-value exploitation. This section is organized along three lines, namely the molecular mechanism of tuber development and formation of high-value components; the major nutritional components; and the influence of cultivar, origin and agronomy on composition.

### 2.1. Biological Basis of Tuber Development and Formation of High-Value Components

Unlike most oil crops, which store reserves in seeds, tiger nut accumulates oil mainly in the underground tuber, a vegetative organ, and therefore represents a rare model for studying oil accumulation in non-seed tissues [9,10]. Assembly of a chromosome-level high-quality genome has provided the resource basis for molecular dissection [11], and establishment of a stable reference gene system has provided the methodological basis for quantifying gene expression [12]. The intensive application of transcriptomics, lipidomics and proteomics in recent years has rapidly advanced our understanding of tuber enlargement and of oil and starch accumulation.

#### 2.1.1. Tuber Initiation and Enlargement

Tuber formation in tiger nut is regulated by photoperiod, hormones and transcription factors acting in concert. RNA-Seq-based transcriptomic studies show that tuber enlargement involves coordinated activation of cell division together with starch and oil accumulation pathways, and multiple transcription factors and hormone signaling pathways have been identified as key regulatory nodes [13]. Integrated anatomical and transcriptomic analyses further revealed the spatiotemporal coordination among vascular differentiation, amyloplast development and oil accumulation during enlargement, and identified a set of candidate genes directly associated with tuber bulking [14]. These results point to molecular targets for improving tuber yield through genetic improvement and cultivation management.

#### 2.1.2. Oil Accumulation and the Regulatory Network of Lipid Metabolism

**Figure 2:**
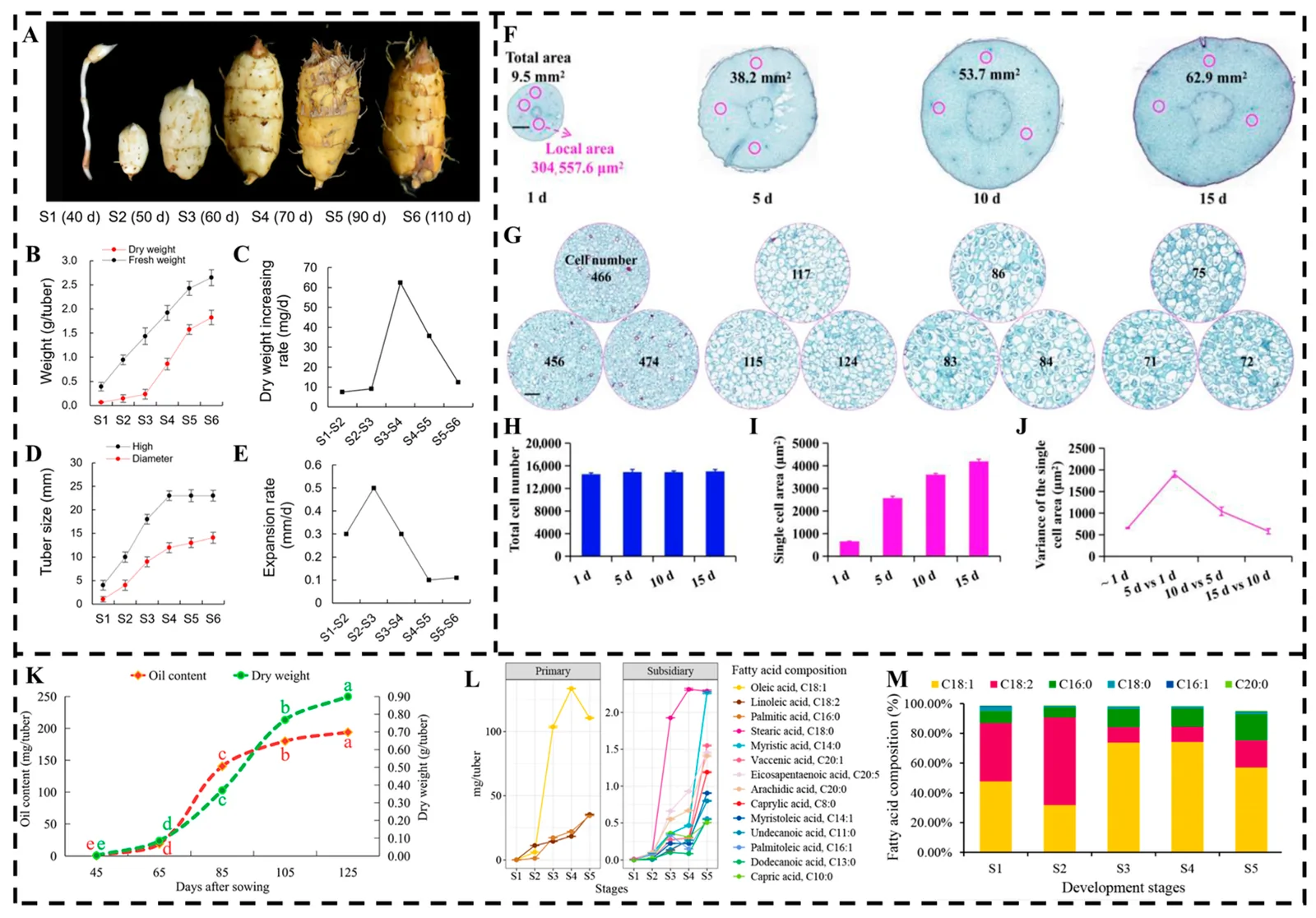
Tuber development and oil accumulation in tiger nut (*Cyperus esculentus* L.): morphological, growth, cytological and lipid-accumulation dynamics. (**A**) Morphological changes in the developing tubers; the horizontal axis denotes six developmental stages, S1–S6, corresponding to 40, 50, 60, 70, 90 and 110 d after sowing [13]. (**B**) Changes in dry and fresh weight per tuber during tuber development [13]. (**C**) Rate of dry weight increase during tuber development [13]. (**D**) Changes in tuber size during tuber development [13]. (**E**) Daily tuber expansion rate during tuber development [13]. (**F**) Entire central transverse section of tubers from 1 d to 15 d; bar = 1000 μm; the red circle marks the local area shown in (**G**) [14]. (**G**) Cell morphology in the fixed local area; bar = 100 μm [14]. (**H**) Estimated total cell number in the transverse section [14]. (**I**) Estimated single-cell area in the transverse section [14]. (**J**) Variation in the growth rate of single-cell area [14]. (**K**) Dry matter and oil accumulation curves of the tuber during development [17]; lowercase letters (a–e) indicate significant differences among developmental stages. (**L**) Accumulation of individual fatty acids across the five developmental stages [17]. (**M**) Proportion of the six most abundant fatty acids in tuber oil [17]. Reused panels are licensed under CC BY 4.0.

Tuber oil is stored mainly in the form of triacylglycerol (TAG). Combined transcriptomic and lipidomic analyses indicate that glycolipids, phospholipids and glycerolipids are markedly enriched during development, while the proportion of phosphatidylcholine (PC) declines continuously and that of phosphatidylethanolamine (PE) declines markedly at early and middle stages, suggesting that PC actively participates in TAG synthesis, whereas PE contributes to TAG metabolism at these stages [15]. Comparative transcriptomics showed that yellow tiger nut has a higher oil accumulation capacity than the related *Cyperus rotundus*, reflecting coordinated up-regulation of genes involved in de novo fatty acid synthesis, TAG assembly and lipid droplet formation [16]. microRNAs have been shown to participate in the post-transcriptional regulation of lipid metabolism and tuber development, adding a further layer to the regulatory network [17], and seed-like proteomic profiling indicates that tiger nut tubers display a seed-like protein composition in which ABI3/WRI1/LEC1-type transcription factors may be involved in desiccation tolerance and high oil accumulation [9]. These multiscale dynamic processes, from morphology and cytology through to lipid accumulation, are summarized in Figure 2.

#### 2.1.3. Key Genes of Oil Biosynthesis and Their Functional Validation

The shift from ‘omics association’ to functional validation represents the most important recent advance (Table 1). The type-II diacylglycerol acyltransferase *CeDGAT2-2* is highly expressed in tubers, and its heterologous overexpression markedly increases the total lipid and oleic acid contents of tobacco leaves [18]. The proteins encoded by two Δ9 fatty acid desaturase genes, *CeSAD1* and *CeSAD2*, act as key enzymes for oleic acid accumulation; *CeSAD1* shows a stronger substrate preference and improves low-temperature tolerance [19], a genome-wide analysis of the SAD family has further extended this insight [20], and systematic identification of the membrane-bound fatty acid desaturase family has enlarged the pool of candidate genes for oil quality improvement [21]. Six oleosin family members were identified, all preferentially expressed in tubers and positively correlated with oil accumulation, and overexpression of *CeOLE2* and *CeOLE5* significantly increased leaf oil content [10]. The transcription factor *CeWRI3* is particularly noteworthy, because it promotes TAG accumulation by activating *CeOLE2* expression, thereby establishing for the first time in tiger nut a complete regulatory chain running from transcription factor through structural gene to oil accumulation [22]; *CePAH1* has also been verified to participate in DAG and TAG metabolism [15]. It should be stressed, however, that most of these validations were obtained in heterologous systems such as tobacco or yeast; the direction of the effect is informative, but its magnitude cannot be assumed to be reproduced to the same extent in tiger nut tubers themselves, and tuber-specific functional validation is therefore still required. These functional validations define the genetic elements available for molecular design breeding of tiger nut.

### 2.2. Major Nutritional Components

The nutritional value of tiger nut rests on a four-in-one composite of oil, protein, starch and dietary fiber, which fundamentally distinguishes it from conventional oilseed or starch crops. Reported compositions vary considerably between studies, and cultivar, origin and cultivation conditions are important sources of this variation in addition to the analytical methods used (Section 2.3). Table 2 places this four-in-one composite against the edible oil crops on the one hand and the starchy root and tuber crops on the other.

Read as a SWOT assessment, the comparison can be summarized as follows. The strengths of tiger nut lie in a single organ that supplies oil rich in oleic acid, together with starch, protein and dietary fiber, and in a fatty acid profile close to that of olive oil. Its weaknesses lie in a composition that varies strongly with cultivar, origin and agronomy; in a protein content of only about 5–10%; and in a health-function evidence base still dominated by in vitro work. The opportunities lie in cultivation on marginal and saline–alkali land and in the cascading biorefinery of its processing residues, while the principal threats are the absence of large-scale agronomic and quality-control systems and the risk of mycotoxin contamination during field and storage phases.

#### 2.2.1. Lipid and Fatty Acid Composition

Tiger nut oil consists mainly of monounsaturated fatty acids, with oleic acid typically accounting for 65–75% of total fatty acids, a profile close to that of olive oil. Its lipid-modulating effect has been verified in animal experiments (Section 3.4) [23], and this high-oleic-acid character underpins its positioning as a healthy edible oil [1]. The Chinese grain industry standard LS/T 3259-2018 *Cyperus esculentus* oil [24] specifies an iodine value of 74–97 g I_2_/100 g oil and a saponification value of 176–196 mg KOH/100 g oil, and lists oleic acid 67.7–74.6%, linoleic acid 8.8–11.5%, palmitic acid 12.2–14.2%, stearic acid 2.4–4.9% and linolenic acid 0.2–1.9% as the main fatty acid indices, values broadly consistent with the ranges reported above. The oil also contains natural antioxidant constituents such as tocopherols and phytosterols, and endogenous hydratable phospholipids have been shown to enhance the oxidative stability of the oil [25]. A recent study further identified 36 endogenous polyphenols of flavonoid type in tiger nut oil, among which quercetin, catechin and eriodictyol showed the strongest antioxidant activity; their comparatively low bond dissociation energy is considered key to maintaining the oxidative stability of the oil [26].

#### 2.2.2. Protein and Its Processing Functionality

Tiger nut protein accounts for about 5–10% of the tuber and has a well-balanced amino acid composition. Compared with soy protein isolate, tiger nut protein shows good emulsifying, foaming and thermal stability, and in vitro fermentation with gut microbiota enriches beneficial bacteria and promotes the formation of short-chain fatty acids, including acetate, propionate and butyrate, indicating its potential as a sustainable plant protein ingredient [27]. In vitro studies also report antioxidant and anti-inflammatory activities for tiger nut protein and its hydrolysates (Section 3.2) [28]. Functional properties such as solubility and emulsification can be further improved by physical treatments, including ultrasound (Section 4.2.2).

#### 2.2.3. Structure and Functional Properties of Starch

The multiscale structure of tiger nut starch, encompassing granule size, amylose content and crystalline type, jointly determines its gelatinization, retrogradation and functional behavior, and it varies significantly with the tuber material and with the history of the meal from which the starch is isolated, so that differences in granular order, crystallinity and amylopectin chain length translate into different thermal transition temperatures, pasting and textural behavior and freeze–thaw stability [29]; its interaction with protein further governs the structural evolution during hot extrusion and the oil absorption of the final product [30]. In particular, the presence of resistant starch lowers the in vitro digestion rate of tiger nut starch, and its content can be further increased by physical-field treatments (Section 4.3.2) [31], which indicates the potential to develop low-GI foods, but does not in itself demonstrate a low glycemic index of the final product. Chemical, enzymatic and physical-field modifications are described in Section 4.3.

#### 2.2.4. Dietary Fiber and Other Functional Components

Tiger nut tubers are rich in dietary fiber; the soluble fraction is dominated by acidic heteropolysaccharides rich in galactose, glucose and arabinose [32], whereas the insoluble fraction shows good water-holding and texture-improving effects in systems such as meat batters (see Section 5.3 for applications) [33]. Fructans have also been detected in tiger nut tubers, where a spectrophotometric determination reported a total fructan content of 13.49%, indicating that the soluble fraction should not be treated as a single polysaccharide class [34]. Functional components like polyphenols [35] and polysaccharides [36] are also present, and the mineral profile is characterized by potassium, magnesium and phosphorus [37]. The combination of dietary fiber and polyphenols endows tiger nut with both high-fiber and antioxidant attributes.

### 2.3. Effects of Cultivar, Origin and Agronomic Practice

Variation in composition is a persistent feature of tiger nut research and industrial practice, and cultivar, origin and agronomic conditions are its main sources. At the cultivar level, starches isolated from different tiger nut materials differ significantly in multiscale structure and functional properties [29], while cytological and ploidy analyses have revealed fertility defects in the main cultivars grown in China that constrain conventional cross-breeding [38]. At the origin level, multimodal identification based on stable isotopes and mineral elements enables high-precision traceability of Chinese tiger nut origins [39], and non-targeted liquid chromatography fingerprints effectively discriminate PDO from non-PDO raw material [3], indicating a systematic influence of origin on the chemical phenotype of the tuber. At the agronomic level, foliar selenium application significantly increases tuber selenium content and improves silage and nutritional quality [40], and the halotolerant endophyte *Franconibacter* sp. YSD YN2 promotes plant growth and enhances salt tolerance, making cultivation on saline–alkali land feasible [41]. The molecular basis of stress tolerance is being resolved in parallel: *CePP2C19* has been found to reduce ABA sensitivity by interacting with the ABA receptor *CePYR1*, and its overexpression enhances drought resistance [42]; the phenylpropanoid and flavonoid biosynthesis pathways of the tuber are strongly activated under drought stress [43]; and the Dof- and LEA_5-domain protein families have been identified as candidate factors in stress response and desiccation tolerance [44]. Disease pressure is a further agronomic constraint, since a newly described sobemovirus has been detected at high incidence in tiger nut fields and causes leaf yellow mosaic, dwarfism and marked tuber yield loss [45]. These studies, however, rarely separate the variance attributable to genotype from that caused by environment and by the analytical methodology used, because cultivar comparisons are often confounded with site and season, and different extraction and determination protocols are applied in different laboratories; the reported compositional ranges should therefore be read as operational rather than intrinsic. These findings indicate that the compositional advantage of tiger nut is not a fixed attribute but the outcome of genotype–environment interaction. This explains why raw materials from different batches behave differently during processing and indicates that cultivar selection and agronomic management are as important as processing technology for value enhancement. In addition, tubers are at risk of contamination with ochratoxin A, zearalenone and aflatoxin B1 in the field and during storage [46]; quality control of raw material should accordingly be moved upstream to cultivation, storage and transport, and linked with end-product testing in a closed loop.

## 3. Bioactive Components of Tiger Nut and Their Health-Promoting Effects

Bioactive components and their health functions provide the core scientific support for the high-value exploitation of tiger nut. This section first surveys the main bioactive components, then groups the available evidence by function and finally evaluates the overall level of evidence.

### 3.1. Major Bioactive Components

The bioactive components of tiger nut can be grouped into four categories. First, the lipophilic fraction is dominated by monounsaturated fatty acids represented by oleic acid, accompanied by tocopherols and phytosterols [1,23]. Second, the phenolic fraction consists mainly of flavonoids and phenolic acids, which are abundant in the tuber and, in particular, in its processing by-products, and constitute the material basis of the antioxidant activity [35,47]; their retention and release are affected by the food matrix and by processing, and thermal treatment, such as roasting, markedly alters the aroma profile, the bioactive peptide complement and the functional properties of the tuber [48]. Third, protein-derived bioactive peptides are released by enzymatic hydrolysis of tiger nut protein and include antioxidant peptides [49,50], α-glucosidase inhibitory peptides [51] and immunomodulatory peptides [52], representing an important route for converting protein into high-value functional peptides. Fourth, within the polysaccharides and dietary fiber group, soluble dietary fiber shows good antioxidant and metal-ion-binding capacities [32], and acetylation further improves the antioxidant and immunomodulatory activities of the polysaccharides [53].

### 3.2. Antioxidant and Cytoprotective Effects

**Figure 3:**
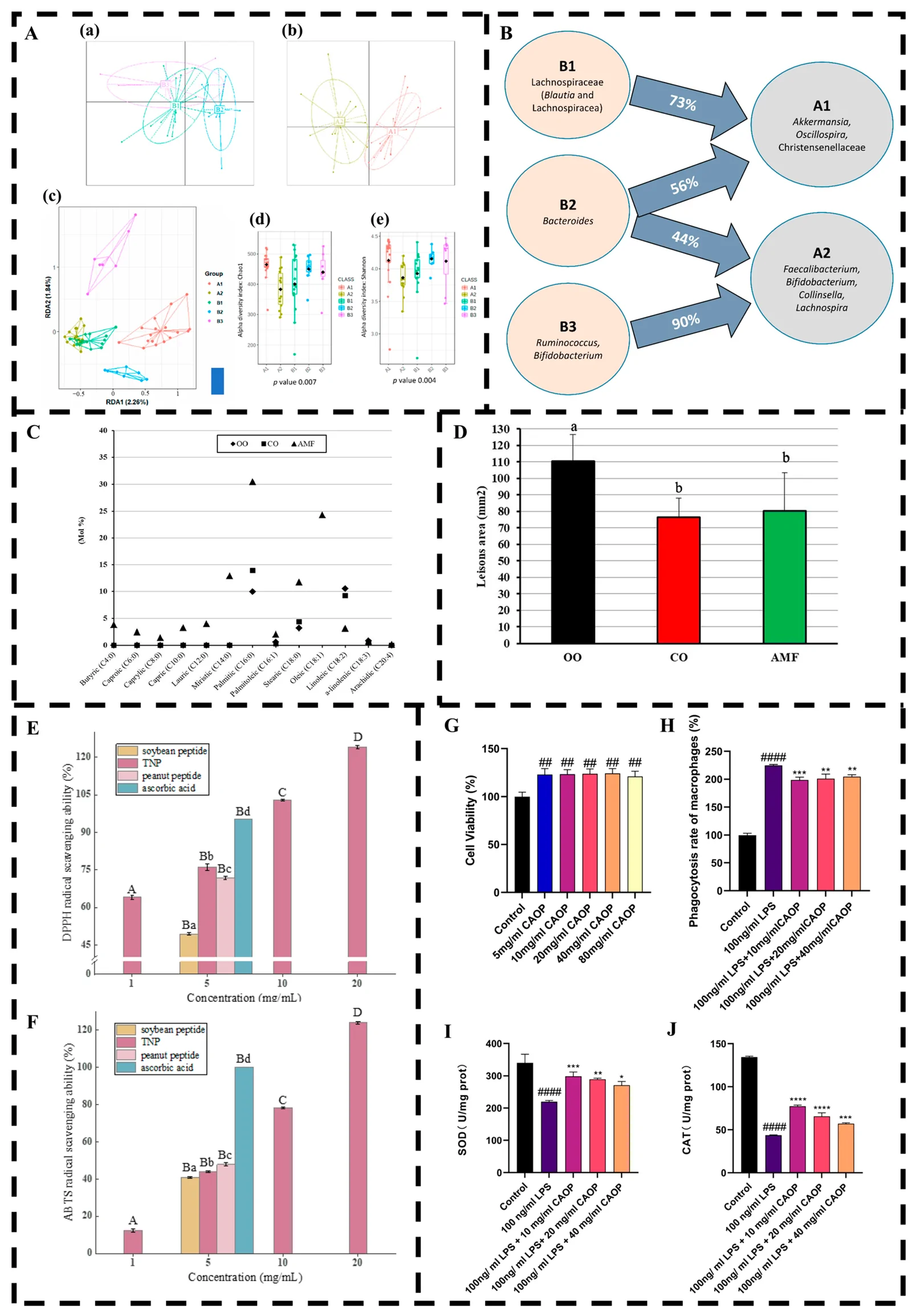
Health effects of tiger nut (*Cyperus esculentus* L.): gut microbiota modulation in a human intervention trial, antioxidant and cytoprotective activity of tuber peptides in vitro, and lipid-related outcomes in a mouse model. (**A**) Microbial clusters and representative microbial taxa at the genus level in the human intervention trial: partitioning around medoid clustering of volunteers into three groups at baseline (**a**) and two groups after intervention (**b**), visualized by principal coordinate analysis; (**c**) redundancy discriminant analysis (RDA) showing distinct microbial communities among clusters; alpha diversity expressed as (**d**) richness (Chao1 index) and (**e**) diversity (Shannon index) [54]. (**B**) Proportion of participants whose microbiota composition migrated from the baseline clusters (B1–B3) to new genus-level profiles (A1–A2) after tiger nut milk (horchata) consumption [54]. (**C**) Fatty acid profile of olive oil (OO), chufa oil (CO) and anhydrous milk fat (AMF) [23]. (**D**) Atherosclerotic lesion area (mm^2^) in the aortic arch of C57BL/6N mice fed olive oil (OO), chufa oil (CO) or anhydrous milk fat (AMF); data are mean ± SE; groups with unlike superscripts (a, b) differ significantly at *p* ≤ 0.05 [23]. (**E**) DPPH• radical scavenging activity of tiger nut peptides (TNPs) compared with soybean peptides, peanut peptides and ascorbic acid at 1–20 mg/mL [50]. (**F**) ABTS•^+^ radical scavenging activity of the same four preparations at 1–20 mg/mL; apparent scavenging values exceeding 100% in (**E**) and (**F**) arise from the colorimetric nature of these assays (sample color and dilution effects) and should not be read as complete or supra-complete radical removal. Different uppercase letters and lowercase letters represent statistically significant differences (*p* < 0.05) [50]. (**G**) Viability of LPS-injured macrophages treated with *Cyperus esculentus* protein (CAP) at 5–80 mg/mL [28]. (**H**) Phagocytosis rate of macrophages under the same treatments [28]. (**I**) Superoxide dismutase (SOD) activity of LPS-injured macrophages [28]. (**J**) Catalase (CAT) activity of LPS-injured macrophages [28] In (**G**–**J**), hash symbols (##, ####) indicate significant differences from the untreated control, and asterisks (*, **, ***, ****) indicate significant differences from the LPS-only group, with a larger number of symbols denoting a higher level of significance. Reused panels are licensed under CC BY 4.0.

The antioxidant activity of tiger nut results from the combined contribution of several classes of components, including polyphenols, bioactive peptides and polysaccharides. Polyphenols scavenge free radicals and inhibit lipid peroxidation in vitro, and the endogenous flavonoids of the oil have been identified as the chemical basis of its oxidative stability (Section 2.2.1) [26,47]; the protein and its peptide fragments show antioxidant effects at both the chemical and cellular levels [28,50] (Figure 3E–J); and acetylated polysaccharides protect macrophages against inflammatory injury by reducing reactive oxygen species and improving cell morphology [53]. The magnitude of these activities is also governed by structural features that are rarely reported in a comparable form—molecular weight, monosaccharide composition, degree of substitution and chain configuration for polysaccharides, and chain length and amino acid sequence for peptides—which currently prevents firm structure–activity relationships from being established. Most of these conclusions, however, derive from in vitro chemical assays or cell-based experiments.

### 3.3. Modulation of Gut Health

Modulation of the gut microbiota is the functional direction supported by the most complete body of evidence for tiger nut. The only human intervention reported so far enrolled 31 healthy adults who consumed 300 mL of natural, unprocessed horchata with no added sugar daily for three days; no consistent change in the microbial profile was detected at the population level, and the shift toward taxa associated with butyrate production became apparent only after the volunteers were stratified by their baseline enterogroup. The study had no separate control arm and lasted only three days, and it reported changes in relative abundance rather than microbial metabolites or clinical endpoints, so the compositional shift alone does not yet demonstrate a physiological benefit [54] (Figure 3A,B). In vitro fermentation studies show that tiger nut protein can be utilized by the gut microbiota and promotes short-chain fatty acid production (Section 2.2.2) [27], while delivery systems built from soluble dietary fiber can modulate the biosynthesis of gut microbial metabolites [55].

### 3.4. Metabolic and Immunomodulatory Effects

With regard to glycemic control, α-glucosidase inhibitory peptides derived from tiger nut meal protein have been screened, and their inhibition mechanism and stability have been characterized [51], and tiger nut starch is digested slowly because of its resistant starch content [56] and has been used to develop products with a low glycemic index [57]. In regard to lipid metabolism, an animal study in C57BL/6N mice showed that dietary tiger nut oil improved cholesterol metabolism and was associated with a lower incidence of gallstone formation than anhydrous milk fat, and that the atherosclerotic lesion area in the aortic arch of the animals receiving chufa oil or anhydrous milk fat was significantly lower than that of the animals receiving olive oil, whereas the two former groups did not differ from each other [23] (Figure 3C,D). In regard to immunity, tiger nut meal peptides [52] and acetylated polysaccharides (Section 3.2) [53] exhibit immunomodulatory activity in cell models, and tiger nut milk shows inhibitory activity against both enveloped and non-enveloped viruses [58], and a fermented tiger nut beverage has been reported to suppress the proliferation of colorectal adenocarcinoma cells in vitro [59]. Most of these functions rest on preliminary evidence obtained at the cellular or animal level.

### 3.5. Grading the Evidence for Health Functions

Taken together, research on the health functions of tiger nut shows a markedly bottom-heavy evidence pyramid (Table 3, Figure 3): physicochemical characterization and in vitro chemical assays dominate (seven studies), cell-based experiments are limited in number (five studies), animal experiments account for only one [23] and only one has reached the level of a human intervention [54]. This means that, apart from gut microbiota modulation, most health claims made for tiger nut lack clinical evidence capable of directly supporting product-level functional claims. The grades assigned in Table 3 follow an explicit, deliberately simple rubric: ‘weak’ denotes evidence based solely on in vitro chemical or enzymatic assays; ‘weak–moderate’ denotes in vitro evidence combined with cell-based experiments; and ‘moderate’ denotes evidence supported by in vivo data together with at least one mechanistic in vitro study. The grading does not incorporate a formal risk-of-bias assessment, because the underlying studies do not report the necessary elements consistently, nor does it weight reproducibility or consistency across studies. The animal study on lipid metabolism is therefore graded ‘moderate’ rather than ‘weak’ because it is an in vivo dietary intervention with a defined comparator fat, a specified mouse strain and reported lipid and lesion endpoints, but it remains a single, unreplicated experiment with seven mice per group, so ‘moderate’ should be read as denoting the type of evidence available rather than a high level of certainty. Future research should therefore shift from asking whether an effect exists to asking at what dose, in what matrix and through which bioavailability route it occurs, giving priority to randomized controlled human intervention trials and to bioavailability data.

## 4. Extraction and Directed Functionalization of High-Value Tiger Nut Components

Processing technology is the bridge between the intrinsic value of a component and its usable functional value. This section reviews the routes for extraction, modification and structural control of the three major components, namely oil, protein and starch, and gathers the emerging physical-field and green extraction technologies by technology type in Section 4.4 (Table 4).

### 4.1. Oil Extraction and Quality Control

#### 4.1.1. Pressing, Refining and Pretreatment

**Figure 4:**
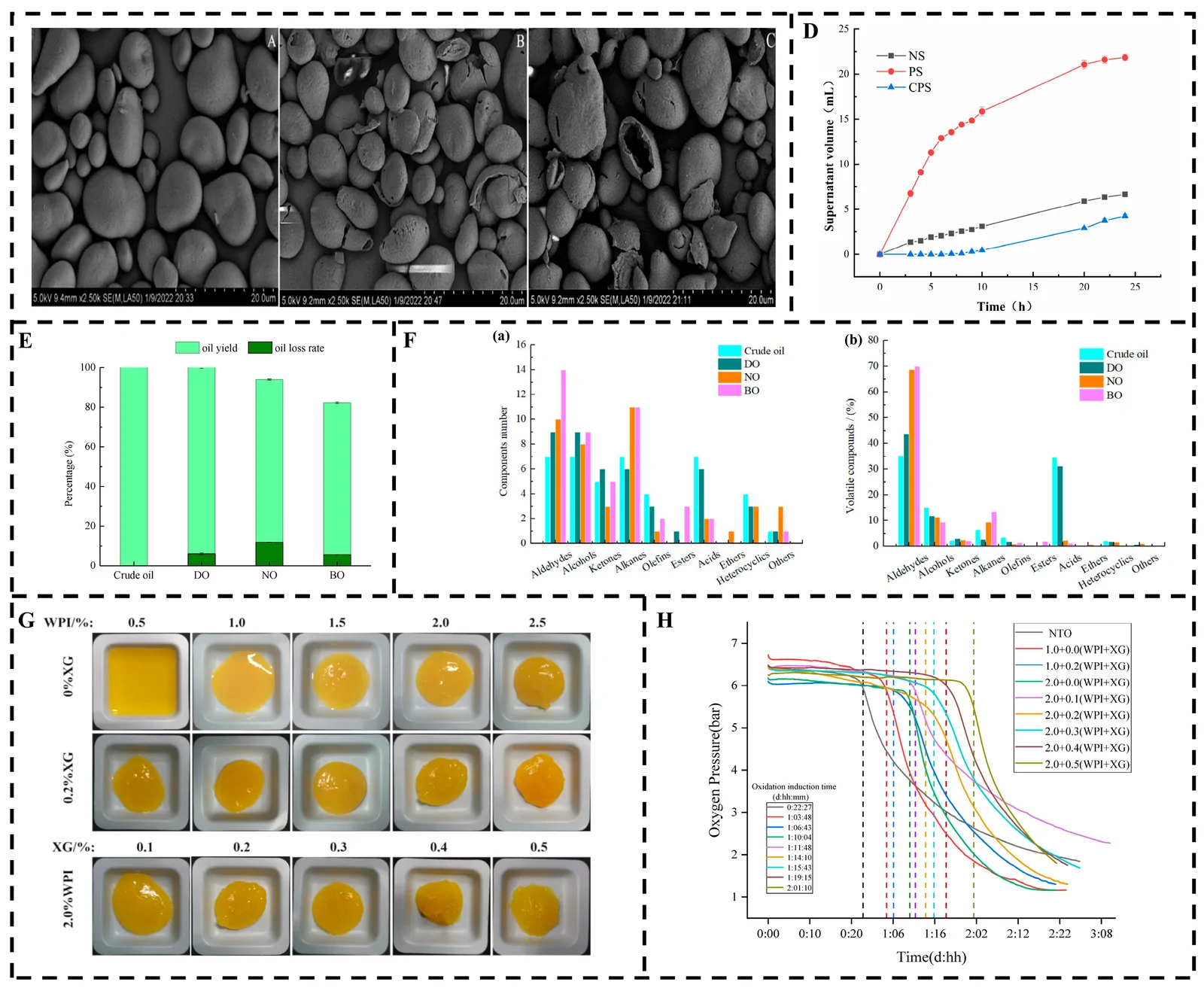
Starch modification and oil refining of tiger nut (*Cyperus esculentus* L.): Retrogradation and microstructure of modified starch, refining yield and flavor, and oleogel morphology and oxidative stability. (**A**) Scanning electron micrograph of native *Cyperus esculentus* starch (NS) [73]. (**B**) Scanning electron micrograph of *Cyperus esculentus* porous starch (PS) [73]. (**C**) Scanning electron micrograph of *Cyperus esculentus* cross-linked porous starch (CPS) [73]. (**D**) Retrogradation curves of NS, PS and CPS [73]. (**E**) Oil yield and oil loss rate of tiger nut oil in different refining processes [60]. (**F**) Changes in the variety and content of volatile compounds in tiger nut oil during refining: (**a**) Number of components and (**b**) volatile compounds [60]. (**G**) Macromorphology of oleogels obtained by templating high internal phase emulsions (HIPEs) stabilized by different concentrations of whey protein isolate (WPI) and xanthan gum (XG) [62]. (**H**) Oxidation stability of tiger nut oil-based oleogels; the intersections of the colored dotted lines with the curves indicate the oxidation induction time [62]. Reused panels are licensed under CC BY 4.0.

Tiger nut oil is produced mainly by pressing followed by refining. Refining removes impurities but simultaneously alters the color and flavor of the oil and the retention of its bioactive constituents [1,60] (Figure 4E,F); cold pressing has therefore attracted attention because it better preserves native bioactives and flavor [60]. The current standard also classifies tiger nut oil into crude and finished grades according to the degree of refining, and sets quality indices for moisture and volatile matter (≤0.5% for crude oil and ≤0.05% for finished oil) and for insoluble impurities (≤0.5% and ≤0.15%, respectively). At the pretreatment end, freeze–thaw treatment disrupts the cellular structure of the tuber and increases oil yield while maintaining good oil quality [61], representing a feasible route toward green and efficient oil recovery.

#### 4.1.2. Structuring of Oil

Oil structuring is an important means of converting liquid oil into solid or semi-solid functional fats. Oleogels based on tiger nut oil have been prepared and their gel strength and storage stability verified [62] (Figure 4G,H); margarine fats low in trans and saturated fatty acids have also been developed [63]; and microencapsulation markedly improves the oxidative stability of tiger nut oil [64]. Together, these technologies extend the application of tiger nut oil in bakery and spreadable products, as well as in delivery systems.

### 4.2. Extraction of Protein and Modification of Its Structure and Functionality

#### 4.2.1. Green Extraction Technologies

Deep eutectic solvents (DESs) and alkaline extraction, followed by acid precipitation, are the two main routes for recovering protein from tiger nut meal; their comparison in terms of structure, functionality and nutritional properties, together with their applicable conditions, is given in Section 6.2 [65]. Because both routes start from the same defatted meal, the choice between them is governed by the intended application of the recovered protein.

#### 4.2.2. Physical Modification

Ultrasound markedly improves the functional properties of tiger nut protein by altering protein structure and surface hydrophobicity; cavitation-generated shear and microstreaming dissociate protein aggregates and partially unfold the polypeptide chain, exposing buried hydrophobic and sulfhydryl groups, and the resulting increase in surface hydrophobicity and molecular flexibility favors adsorption at the oil–water interface. In the study concerned, tiger nut protein dissolved at 20 mg/mL in phosphate buffer (0.2 mol/L, pH 7.0) was treated with a 0.636 cm ultrasonic probe at 200, 300, 400, 500 and 600 W, corresponding to sound intensities of 699.34–2131.25 W cm^−2^, for 30 min in pulsed mode (5 s on, 3 s off), while the sample was maintained at 30–35 °C; protein solubility rose from 41.29% to 67.53%, accompanied by simultaneous improvements in emulsifying activity and emulsion stability. Because only the power was varied while the other conditions were held constant, power alone does not fully account for the effect; since ultrasonic frequency, treatment time and sample temperature jointly govern the extent of cavitation and the degree of unfolding, these parameters are reported inconsistently across studies, and the frequency itself was not reported in the study concerned [66]. Microwave and water-bath heat treatments modify the structure of the alkali-soluble glutenin fraction of tiger nut seed protein—one of the four Osborne fractions of that protein (albumin, globulin, gliadin and glutenin, in which albumin predominates) and chemically unrelated to the wheat storage protein of the same name—and thereby improve its functional properties and in vitro gastrointestinal digestibility [67].

#### 4.2.3. Enzymatic Cross-Linking and Glycation

Enzymatic cross-linking and glycation improve the emulsifying performance of tiger nut protein by covalently modifying the interfacial layer: TGase catalyzes acyl transfer between the γ-carboxamide group of peptide-bound glutamine and the ε-amino group of lysine, forming inter- and intramolecular ε-(γ-glutamyl)lysine cross-links that raise the molecular weight and the viscoelasticity of the adsorbed film, whereas, in glycation, the covalently attached carbohydrate moiety increases steric stabilization and shifts the isoelectric point of the protein. Among these routes, transglutaminase (TGase)-induced glycation yields protein hydrolysates with high emulsifying performance [68], whereas TGase-cross-linked tiger nut protein–whey protein complexes have been used as Pickering stabilizers [69] (see Section 5.5 for applications).

#### 4.2.4. Construction of Protein–Polyphenol Composite Structures

Protein–polyphenol complexation provides a route to multifunctional interfacial materials. Structure–activity studies show that the galloyl group is the key structural unit governing the binding strength between tiger nut protein and tea polyphenols, because the larger number of phenolic hydroxyls available for hydrogen bonding and hydrophobic association with the protein increases the binding affinity and the number of occupied sites; the resulting change in the surface charge and hydrophobicity of the protein underlies the improved interfacial behavior of the complexes [70], and physical modifications, including ultrasound, microwave and heat treatments, strengthen the non-covalent binding between protein and proanthocyanidins and improve the properties of the complexes [71]. The resulting tiger nut protein/tea polyphenol composite nanoparticles increase the stability of anthocyanins in Pickering emulsions [72], combining the interfacial properties of the protein with the antioxidant activity of the polyphenols.

### 4.3. Starch Extraction, Modification and Control of Digestibility

#### 4.3.1. Isolation Technologies

Tiger nut starch can be isolated from defatted meal, and its yield, physicochemical properties and in vitro digestibility are determined jointly by the raw material and the process; microwave treatment enables efficient isolation of starch from defatted meal [56].

#### 4.3.2. Chemical/Enzymatic and Physical-Field Modification

Enzymatic modification combined with sodium phytate cross-linking alters the granular structure, gelatinization and retrogradation behavior of the starch [73] (Figure 4A–D), whereas modification with octenyl succinic anhydride (OSA) yields amphiphilic starch nanoparticles and broadens their interfacial applications [74] (see also Section 5.5). Among physical fields, dielectric barrier discharge plasma (DBDP) and microwave treatment promote the complexation of starch with epigallocatechin gallate (EGCG), increasing the resistant starch content by 6–9% and slowing the digestion rate [31].

#### 4.3.3. Regulation Through Multicomponent Complexation

Interactions between starch and other components markedly affect the viscosity, rheology and microstructure of the system; blending with sanxan gum produces a synergistic thickening effect [75], hyaluronic acids of different molecular weights allow directed control of gelatinization and digestion behavior [76], and starch–flavonoid interactions lower in vitro digestibility [77]. These results support the use of complexation as a means of achieving directed control of digestion properties.

### 4.4. Emerging Physical-Field and Green Extraction Technologies

Beyond modifications aimed at individual components, a group of intensification-oriented processing technologies has emerged in recent years (Table 4); their ‘green’ character and thermal load, however, differ markedly and must be judged case by case. Microwave technology, valued for its high heating efficiency and strong penetration, has been used to improve the functionality and storage stability of tiger nut flour [78], to construct starch–polyphenol complexes [31] and to recover antioxidant components from by-products with high efficiency [6]. Radio-frequency drying preserves the functional properties of tiger nut flour better than conventional drying [79], whereas combined infrared–microwave baking has been used for process optimization of products such as biscuits [80]. Ultra-high-pressure homogenization, a typical non-thermal technology, retains volatile flavor compounds in tiger nut beverages better than conventional thermal pasteurization [81]. Ultrasound is the most widely applied of these technologies; besides improving protein functionality [66], it can regulate protein–polyphenol binding through three physical modification modes [71]. With respect to enhanced mass transfer and pretreatment, steam explosion disrupts the compact structure and increases enzyme accessibility, significantly enhancing the enzymatic digestibility of the tuber [82], whereas deep eutectic solvents replace conventional acid–alkali systems as green solvents for extracting components such as protein [65] (Section 6.2 gives details). It should be stressed that the label ‘green’ needs qualification: microwave and radio-frequency treatments rely on dielectric heating and steam explosion combines high temperature with high pressure, so the thermal exposure involved depends on the treatment intensity, and the energy demand, solvent recovery and life-cycle performance of these processes have not been quantified in a comparable way. Overall, these technologies are moving from isolated optimization toward combined application; however, the depth of comparative process evaluation remains limited (Section 7.2).

**Table 4:** Summary of processing technologies applied to tiger nut and their key outcomes.

Technology	Target	Key Results	References
Cold pressing/refining	Oil	Cold pressing better retains flavor and bioactives; refining alters color and flavor	[1,60]
Freeze–thaw pretreatment	Oil	Disrupts cell structure, increases oil yield and maintains quality	[61]
Oleogel/margarine/microencapsulation	Oil	Achieves oil structuring; microencapsulation markedly improves oxidative stability	[62,63,64]
Ultrasound (0–600 W)	Protein	Solubility 41.29% → 67.53%, with parallel improvement in emulsification	[66]
Microwave/water-bath heating	Glutenin (alkali-soluble fraction) in meal	Improves structure, functional properties and in vitro gastrointestinal digestibility	[67]
TGase cross-linking/glycation	Protein	Produces hydrolysates and complexes with high emulsifying performance	[68,69]
Deep eutectic solvents/alkaline extraction–acid precipitation	Meal protein	Green extraction; distinct advantages in structure and functionality	[65]
Enzymatic modification + sodium phytate cross-linking	Starch	Alters granular structure, gelatinization and retrogradation behavior	[73]
OSA modification	Starch	Produces amphiphilic starch nanoparticles	[74]
DBDP/microwave physical fields	Starch	Promotes starch–EGCG complexation; resistant starch increases by 6–9%	[31]
Microwave treatment of defatted meal	Starch	Efficient isolation of starch	[56]
Ultra-high-pressure homogenization	Plant-based beverage	Retains volatile flavor better than conventional thermal pasteurization	[81]
Radio-frequency drying	Tiger nut flour	Better retention of functional properties	[79]
Combined infrared–microwave baking	Biscuits	Optimizes process, texture and nutritional properties	[80]
Steam explosion	Tuber/biomass	Disrupts compact structure and markedly increases enzymatic digestibility	[82]
Microwave-assisted extraction	Polyphenols in by-products	Efficient recovery of antioxidant components	[6]

## 5. Development of High-Value Tiger Nut Foods

The high-value exploitation of tiger nut is ultimately realized in the construction of diverse food systems. This section is organized around product categories (Table 5).

### 5.1. Plant-Based Beverages and Fermented Foods

**Figure 5:**
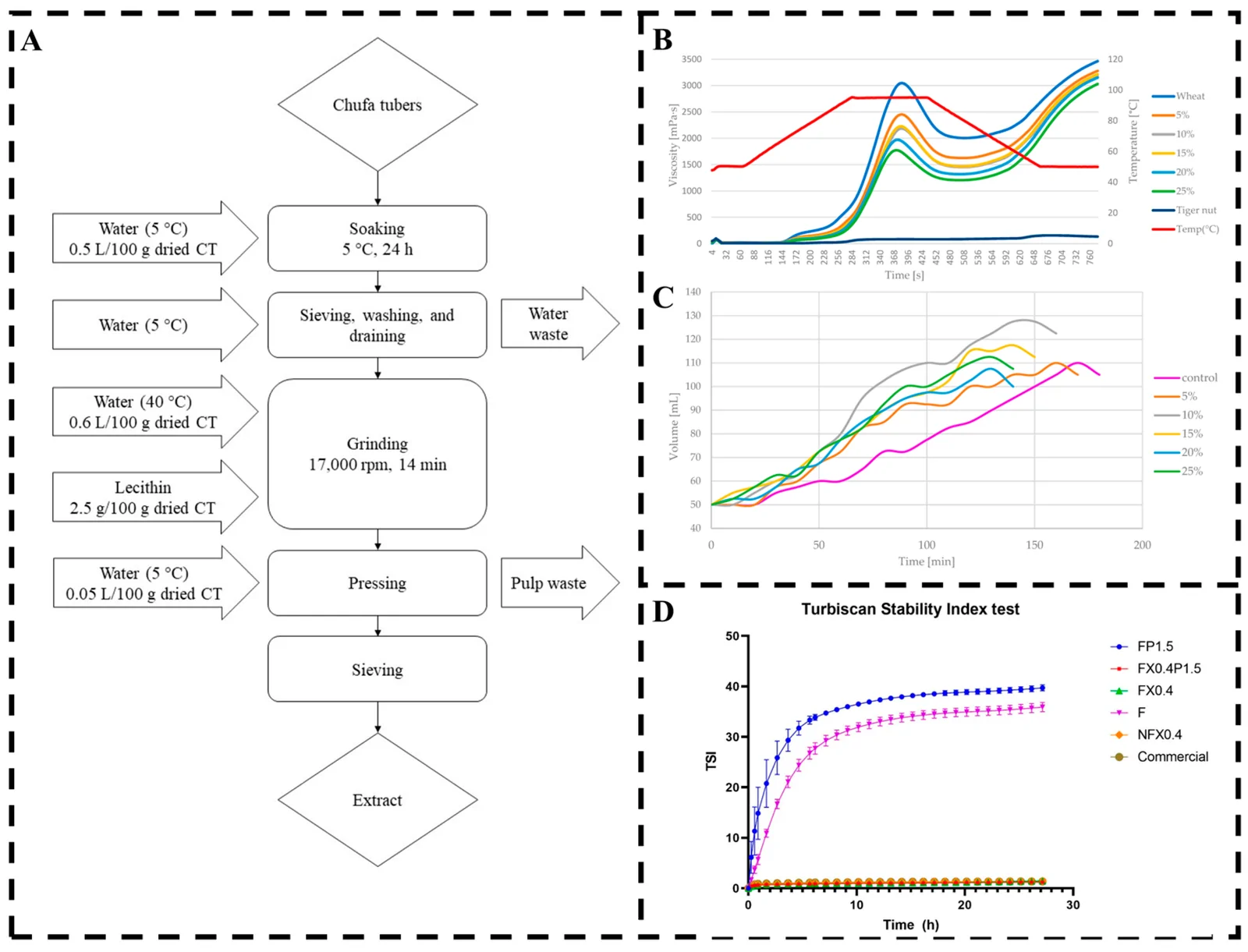
Tiger nut as a food ingredient: processing and physical stability of tiger nut milk (horchata), and pasting and dough-rising performance of wheat–tiger nut blends. (**A**) Flowchart of the production process for the chufa-based liquid extract [85]. (**B**) Pasting curves of wheat–tiger nut blends at 5–25% tiger nut flour addition; means followed by different lowercase letters are statistically different (*p* = 0.05) [90]. (**C**) Dough-rising characteristics of the control and wheat–tiger nut blends at 5–25% tiger nut flour addition [90]. (**D**) Turbiscan stability index measured over 27 h at 5 °C of non-fermented tiger nut milk with 0.4% xanthan gum (NFX0.4), fermented tiger nut milk with 0.4% xanthan gum (FX0.4), fermented tiger nut milk with 0.4% xanthan gum and 1.5% potato protein (FX0.4P1.5), fermented tiger nut milk with 1.5% potato protein (FP1.5), fermented non-formulated tiger nut milk (labelled F in the legend), and a commercial cold buttermilk soup; data are means of triplicates [85]. Reused panels are licensed under CC BY 4.0.

Tiger nut milk, traditionally known as horchata de chufa, represents the most mature food format of tiger nut. Its sensory attributes, volatile composition and consumer acceptance have been systematically studied, and PDO and non-PDO raw materials differ significantly in sensory and volatile profiles, thus providing a basis for quality grading and geographical-indication protection [3,83]. Processing markedly affects beverage quality (Figure 5A,D); non-thermal processing retains volatile flavor compounds better than conventional thermal pasteurization [81], and the technical comparison is given in Section 4.4. The addition of stabilizers and maltodextrin determines the physicochemical and functional properties of the spray-dried powder [84], converting the beverage into an ingredient form convenient for storage and transport. In the fermentation direction, tiger nut beverages are a good plant-based fermentation substrate [85], and the plant-based yoghurt prepared from them performs well in physicochemical, microbiological and nutritional evaluations and can serve as a substitute for conventional dairy yoghurt [86].

### 5.2. Bakery and Cereal Products

In bread systems, the addition of tiger nut flour increases dietary fiber and polyphenol contents, as well as antioxidant activity [87], and the best specific volume and porosity are obtained at a 10% addition level [88]; higher addition levels alter dough rheology and reduce specific volume, whereas moderate germination mitigates this negative effect [89]. For soft wheat–tiger nut composite flours (5–25%), antioxidant activity increases with the addition, level while the decrease in final viscosity remains below 15% [90] (Figure 5B,C). For biscuits and gluten-free products, tiger nut-based gluten-free biscuits show a low glycemic index and a favorable nutritional composition [57], and combined infrared–microwave baking further optimizes their texture and quality [80]. Among cereal products, hydrocolloids improve the structure and cooking quality of fiber-enriched pasta made from tiger nut flour and durum wheat semolina. Addition level, formulation and storage behavior are, however, rarely studied together: the effect of tiger nut flour on loaf volume and crumb structure depends on its proportion in the composite flour and on the water and hydrocolloid balance of the formulation, and starch retrogradation and polyphenol loss during storage can partly offset the nutritional gain achieved at the point of baking. Sensory acceptance is reported in only a few of these studies, and the great majority of the products described in this section are laboratory or pilot prototypes rather than formulations validated at industrial scale or already commercialized, so the transfer of the reported nutritional improvements to marketed bakery products still has to be demonstrated [91].

### 5.3. Meat Products and Fat Replacement

Insoluble dietary fiber from tiger nut can replace part of the fat in meat batters, improving product quality and processing functionality while increasing in vitro protein digestibility [33]; the residue remaining after supercritical CO_2_ defatting of tiger nut milk has been used in pork burgers as a sustainable fat replacer that improves product quality [92] (Section 6.3 gives details); and tiger nut protein improves the gel properties and 3D printability of reduced-sodium chicken batters [93].

### 5.4. Functional and Nutritional Foods

The production process for a meal replacement powder based on rice bran and tiger nut meal has been systematically optimized [94]; tiger nut biscuits supplemented with *Lacticaseibacillus rhamnosus* SL42 (*formerly Lactobacillus rhamnosus*) can serve as a probiotic carrier [95]; and foliar selenium application allows the production of selenium-enriched functional raw material [40]. Breads enriched with tiger nut and *Moringa oleifera* flour have also been reported to improve endothelial function in a hypertensive model by restoring nitric oxide levels and inhibiting arginase and angiotensin-converting enzyme activities [96].

### 5.5. Food Colloids and Delivery Systems for Bioactives

Colloidal and delivery systems are among the fastest-growing directions in recent years, and their structural basis is described in Section 4.2 and Section 4.3. Tiger nut protein–polyphenol composite nanoparticles (Section 4.2.4) can be used to stabilize anthocyanins in Pickering emulsions [72]; TGase-cross-linked tiger nut protein–whey protein complexes (Section 4.2.3) act as Pickering stabilizers that reduce ice crystal size and markedly retard ice-cream melting [69]; water-in-oil-in-water (W/O/W) multiple emulsions stabilized by tiger nut starch can be used for the encapsulation and delivery of lycopene [97]; and OSA-modified starch nanoparticles (Section 4.3.2) provide a further carrier option for Pickering emulsions and interfacial stabilization [74]. These systems offer carrier options for stabilizing and delivering functional bioactives.

**Table 5:** Summary of tiger nut-based food application systems.

Application System	Representative Products	Key Components/Functional Positioning	References
Plant-based beverages	Tiger nut milk (horchata), UHPH beverage, spray-dried powder	Whole matrix; flavor retention and powder stability	[81,83,84]
Fermented foods	Fermented dessert, plant-based yoghurt	Substrate for probiotic fermentation	[85,86]
Bakery products	Bread, biscuits, gluten-free biscuits	Composite flour; dietary fiber and antioxidant fortification	[57,87,88,89]
Cereal products	High-fiber pasta	Hydrocolloids regulate structure and cooking quality	[91]
Meat products	Low-fat meat batter, pork burger, 3D-printed chicken gel	Insoluble dietary fiber, defatted residue, protein	[33,92,93]
Functional and nutritional foods	Meal replacement powder, probiotic biscuits, selenium-enriched products	Dietary fiber, probiotic carrier, selenium fortification	[40,94,95]
Colloids and delivery systems	Pickering emulsions, ice cream, multiple emulsions	Protein–polyphenol particles, starch-based stabilizers	[69,72,97]

## 6. Valorization and Recycling of Tiger Nut-Processing Residues

The valorization and recycling of processing residues represent the point at which the twin goals of high-value exploitation and food-system sustainability converge.

### 6.1. Generation and Resource Characteristics of Residues

**Figure 6:**
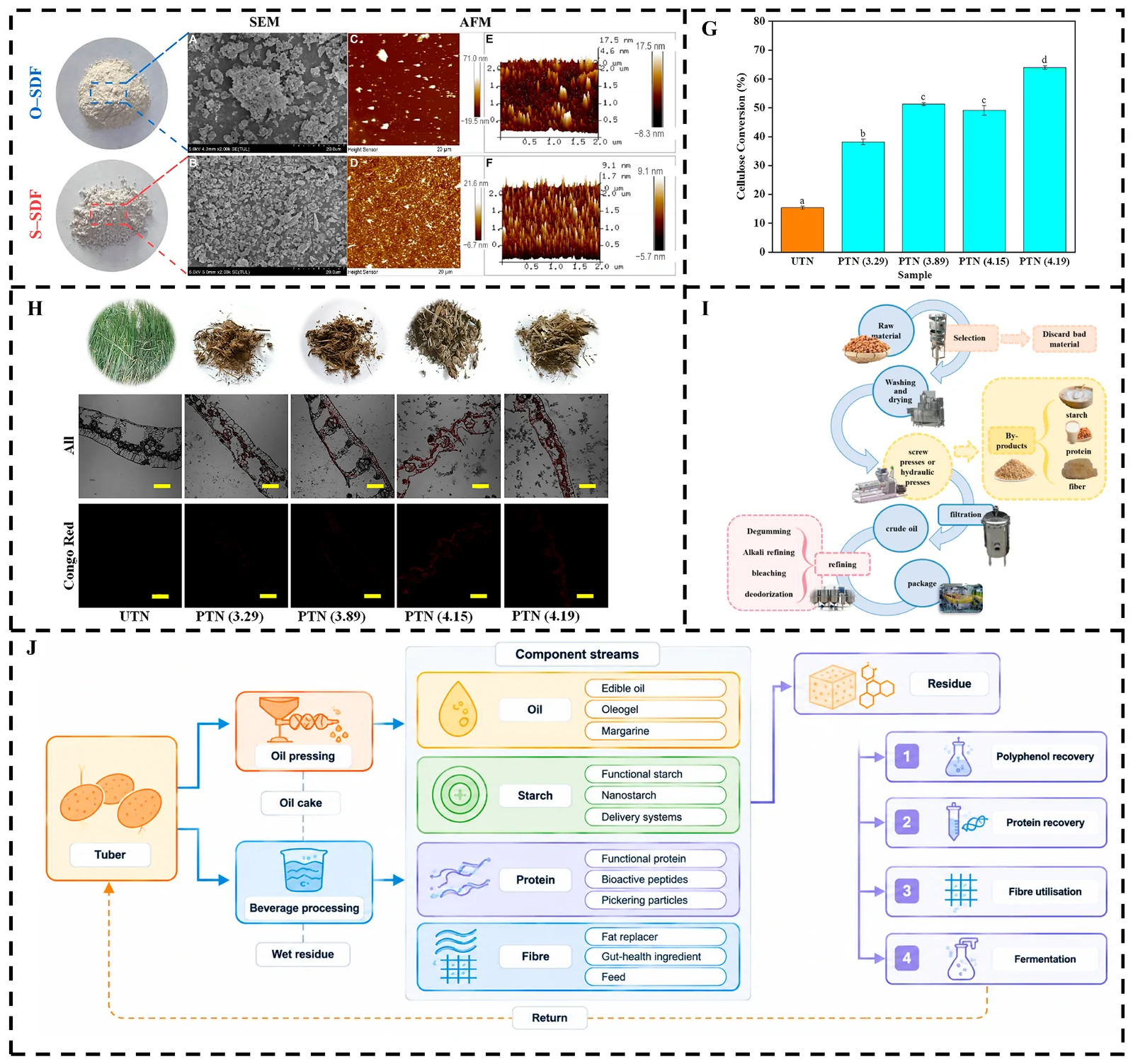
Valorization of tiger nut-processing residues: microstructure of soluble dietary fiber, steam explosion pretreatment and enzymatic digestibility, by-product streams of oil production, and a cascading biorefinery route. (**A**,**B**) Scanning electron microscopy (SEM) images showing the surface morphology of soluble dietary fiber (SDF) prepared from tiger nut residue [32]. (**C**–**F**) Atomic force microscopy (AFM) images of SDF, complementing the SEM observations at higher resolution [32]. (**G**) Cellulose enzymatic hydrolysis performance of tiger nut before and after steam explosion (SE) pretreatment; bars with different superscripts differ significantly (*p* < 0.05, Duncan’s multiple range test) [82]. (**H**) Photographs (top) and confocal laser scanning microscopy (CLSM) images of tiger nut before and after SE pretreatment; cellulose labeled with Congo red appears red; scale bar = 100 μm [82]. (**I**) Preparation of tiger nut oil in the food industry and the by-products generated [4]. (**J**) Proposed cascading biorefinery route for tiger nut residues: the tuber is first split by oil pressing and beverage processing into oil, starch, protein and dietary fiber streams, and the remaining residue is upgraded stepwise through polyphenol recovery, protein recovery, fiber utilization and fermentation, whose output is returned to the tuber chain (author’s own drawing). Reused panels are licensed under CC BY 4.0.

Tiger nut processing generates residues mainly through two routes, namely oil meal (press cake or defatted meal) from oil extraction and wet residue (pomace) from the production of plant-based milk and beverages. Estimated from a tuber oil content of 20–36% (dry-weight basis), oil meal accounts for about 65–80% of the raw material mass after oil extraction. That is, processing 1 t of tubers generates roughly 0.65–0.80 t of oil meal, making it the largest residue stream by quantity (Figure 6I). Such streams are also amenable to physical pretreatment, since steam explosion disrupts the compact structure of the biomass and markedly increases its enzymatic digestibility [82] (Figure 6G,H). Both types of residue still contain abundant usable components, but their composition varies with the process used, since solvent-extracted meal has the highest starch content; distiller’s dried grains with solubles (DDGSs) have the highest crude protein content; and conventional tiger nut meal has the highest crude fiber, neutral detergent fiber and acid detergent fiber contents [98]. Utilization routes therefore have to be designed separately for each process type. The wet residue obtained from horchata production contains up to 59.71 g/100 g total dietary fiber, of which 99.8% is insoluble, together with a water-holding capacity of 8.01 g/g, an oil-holding capacity of 6.92 g/g and good emulsifying capacity and emulsion stability [99]. In addition, pomace can be further processed into soluble dietary fiber [32] and used in the fermentation of blended fruit wines [100], whereas the residues themselves remain an important source of polyphenols [35]. Residues should therefore not be regarded as waste, but incorporated into industrial design as a secondary resource.

### 6.2. Recovery of High-Value Components from Residues

Component recovery from residues can be organized into four routes; because the underlying processes are described above, only the residue-specific aspects are summarized here. For protein recovery, the two established routes are deep eutectic solvent extraction and alkaline extraction followed by acid precipitation, which are compared in Section 4.2.1 and selected according to the intended application [65]. For dietary fiber, soluble dietary fiber recovered from residues combines antioxidant activity with metal-ion-binding capacity (Section 3.1), which makes it suitable as a functional ingredient [32] (Figure 6A–F), whereas the insoluble fraction serves as a fat replacer (Section 5.3) [33]. For polyphenols, microwave-assisted extraction is the route used to recover antioxidant components from residues (process details in Section 4.4) [6], and the individual phenolic compounds present in these residues and the factors governing their recovery have been characterized in detail [35]. Peptide development adds the greatest value, because hydrolysis of residue protein gives the same three functional peptide classes [49,50,51,52], whose activity characterization and mechanisms are presented in Section 3.1 and Section 3.4.

### 6.3. Food Applications of Residues

Residues have been used directly or indirectly in a range of foods, including a meal-replacement powder based on rice bran and tiger nut meal (Section 5.4) [94]; the residue of tiger nut milk remaining after supercritical CO_2_ defatting, used as a sustainable fat replacer in pork burgers that improves product quality while lowering the carbon footprint [92]; a novel non-centrifugal sugar prepared from tiger nut meal, with physicochemical and nutritional characteristics distinct from those of cane sugar [101]; and a wine fermented from a pomace–raspberry blend, whose flavor dynamics have been resolved by combining UHPLC-OE-MS with HS-SPME-GC-MS [100].

### 6.4. Circular Bioeconomy and Cascading Biorefinery

Within the framework of the circular bioeconomy, residue utilization should move from single-component extraction toward cascading utilization of all components. With the tuber at the center, oil flows into edible oil, oleogels and margarine; starch into functional starch, nanostarch and delivery systems; protein into functional protein, bioactive peptides and Pickering particles; and dietary fiber into fat replacers and gut health ingredients. The remaining residue is then subjected to polyphenol extraction, protein recovery and fiber utilization, and finally to fermentation, forming the cascading biorefinery route ‘oil → protein → polyphenols → fiber → fermentation’ (Figure 6J). This route has so far been validated mainly in terms of technical feasibility, while its economic performance and environmental impact have not yet been systematically assessed.

## 7. Conclusions and Perspectives

### 7.1. Main Conclusions

Tiger nut is a composite food resource combining oil, protein, starch and dietary fiber. Its combined nutritional and agronomic advantages give it the potential to move from a regional traditional food to a global high-value food ingredient. Taken together, three main conclusions can be drawn: (1) Building on the assembly of a chromosome-level genome, the cloning and functional validation of genes like *CeDGAT2-2*, *CeSAD1/2*, *CeOLE2*, *CeWRI3* and *CePAH1* have advanced research on the high-oil trait of tiger nut from ‘omics association’ to the level of defined genetic elements, providing clear targets for the molecular improvement of oil quality. (2) The evidence for health functions still rests mainly on in vitro chemical assays and cell experiments, with only one animal study and one human intervention (Table 3), so that, apart from gut microbiota modulation, most health claims remain insufficient to support product-level functional claims. (3) Processing and application are linked mainly at the level of laboratory prototypes: upstream green extraction, physical-field treatment and enzymatic modification allow the composition and functionality of individual components to be steered, and downstream systems have been demonstrated for plant-based beverages such as horchata, bakery products, fat replacement in meat products and colloidal delivery, yet most of these remain at laboratory or pilot scale and only horchata-type beverages and refined tiger nut oil have reached commercial maturity, so that the critical gap between the two ends still lies in the cascading biorefinery of residues and its economic assessment. In summary, the core of high-value tiger nut exploitation lies not in the deep development of any single component but in opening up the complete value chain running from resource through components, function, processing and product to recycling.

### 7.2. Challenges and Future Perspectives

Viewed in terms of research paradigm, most current work still remains within the linear mode of ‘extraction → measurement → activity evaluation’; what the components are has been answered reasonably well, whereas why they are effective remains unclear. Future work should shift toward structure–activity research along the line of ‘structure → interaction → bioavailability → physiological function’. For polyphenols, the quantitative approach centered on ‘structural feature—bond dissociation energy—antioxidant potency’ can be followed; for polysaccharides, acetylation has been confirmed to markedly increase activity, and quantitative relationships between the degree and site of substitution and activity should be established; for bioactive peptides, studies on the structure–activity relationship and stability of α-glucosidase inhibitory peptides provide a reference paradigm. In terms of health functions, priority should be given to randomized controlled human intervention trials, preferably in the gut microbiota direction where preliminary human evidence already exists, and bioavailability studies should be carried out systematically to avoid drawing inferences when effective concentrations in vitro far exceed those achievable in vivo.

On the processing side, existing studies are mostly comparisons of single factors or of a few levels, and they rarely characterize multifactor interactions and process mechanisms. Synergistic multi-physical-field treatment has been shown to markedly increase resistant starch content in starch–polyphenol composite systems, and this concept can be extended to other component systems; near-infrared spectroscopy combined with chemometrics has enabled rapid determination of the nutritional components of tiger nut, and its combination with machine learning and interpretability analysis makes online prediction of raw material grading and process parameters possible. Replacing organic solvents with deep eutectic solvents and replacing conventional thermal treatments with non-thermal processing are clear green directions, but solvent recovery, energy consumption and life-cycle assessment must be conducted in parallel. At the level of standards and regulation, the European Union has completed the safety assessment of tiger nut oil as a novel food under Regulation (EU) 2015/2283 [102], and Valencian horchata is protected by a PDO geographical indication. China has established the rudiments of a standard system centered on the grain industry standard LS/T 3259-2018 *Cyperus esculentus* oil [24] and supplemented by the group standards T/CCOA 66-2023 *Cyperus esculentus* powder [103] and T/CCOA 72-2023 Technical specification for the production of *Cyperus esculentus* oil [104], whereas elements covering raw material grading, components such as protein and starch, functional evaluation and residue utilization are still missing. In addition, fertility defects in the main cultivars limit progress in conventional breeding, whereas the exploitation of halotolerant endophyte resources and the identification of drought-resistance genes create conditions for cultivation on marginal land. Cascading utilization of residues has so far been validated only in terms of technical feasibility and lacks systematic economic accounting and environmental impact assessment; future work should evaluate technical feasibility alongside economic feasibility and sustainability so as not to stop at the laboratory-validation stage. This review also has limitations. The literature search was restricted to English-language peer-reviewed sources indexed in the databases listed above, so studies published in other languages, in the grey literature or in patents were not captured; the available evidence is concentrated in a limited number of research groups and regions, which may bias the reported compositional ranges; few of the original studies report cultivar, growing environment and analytical protocol together, so formal meta-analytical synthesis was not possible; and the health-function evidence remains too heterogeneous in dose, matrix and endpoint to be pooled quantitatively. These limitations are the reason why the grades in Table 3 are descriptive rather than probabilistic.

## Figures and Tables

**Table 1:** Key genes and regulators involved in lipid biosynthesis in tiger nut.

Gene/Regulator	Main Function	Functional Validation or Key Evidence	Reference
*CeDGAT2-2*	Encodes a type-II diacylglycerol acyltransferase that catalyzes the terminal step of TAG synthesis	Heterologous overexpression in tobacco increased total lipid and oleic acid contents	[18]
*CeSAD1*/*CeSAD2*	Encodes Δ9 fatty acid desaturases that catalyze oleic acid formation	Highly expressed in tubers; *CeSAD1* shows stronger substrate preference and improves low-temperature tolerance	[20]
*CeOLE2*/*CeOLE5*	Encodes oleosins that stabilize lipid droplet structure	Six members identified in the family; overexpression increased leaf oil content in tobacco	[10]
*CeWRI3*	Encodes a WRI-type transcription factor	Activates *CeOLE2* expression and promotes TAG accumulation	[22]
*CePAH1*	Encodes a phosphatidic acid phosphohydrolase involved in DAG and TAG metabolism	Induced expression in a yeast system increased DAG and total lipid	[15]
miRNA (multiple members)	Non-coding small RNAs that post-transcriptionally regulate lipid metabolism and tuber development	Identification of the miRNAs and their target gene networks	[17]
Chromosome-level genome	Reference genome resource for dissecting oil and starch traits	High-quality assembly completed	[11]

**Table 2:** Comparison of tiger nut with major edible oil crops and with major starchy root and tuber crops.

Crop (Edible Part)	Oil Content (% Dry Weight)	Oleic Acid (% of Total Fatty Acids)	Protein (g/100 g)	Dietary Fiber (g/100 g)	References
Tiger nut (*Cyperus esculentus*, tuber)	20–36	65–75	5–10	9–15	[7,8]
Soybean (seed)	18–24	15–36	36.5	9.3	[7]
Rapeseed (seed)	37.5–46.3	56.0–72.0	18.4–21.5	12.8	
Peanut (seed)	46–57	37.0–55.6	25.8	8.5	
Sunflower (seed)	46–50	16.4–27.6	20.8	8.6	
Olive (fruit)	31–56	54.5–80.2	0.8	2.4	
Potato (tuber)	0.4	3	1.7	2.4	[8]
Sweet potato (root)	0.2	5	1.6	3.0	
Cassava (root)	0.7	30	1.4	1.8	
Yam (tuber)	0.7	10	1.5	4.1	

**Table 3:** Summary of evidence levels for the health functions of tiger nut.

Functional Category	Representative Components	Main Type of Evidence	Strength of Evidence	References
Antioxidant activity	Polyphenols, bioactive peptides, acetylated polysaccharides	In vitro chemical assays, cell experiments	Weak–moderate	[26,47,53]
Gut microbiota modulation	Whole beverage, dietary fiber, protein	Human intervention, in vitro fermentation	Moderate	[27,54,55]
Glycemic control	α-Glucosidase inhibitory peptides, resistant starch	In vitro enzymology, in vitro digestion	Weak	[51,56,57]
Lipid metabolism regulation	Tiger nut oil	Animal experiment (mice)	Moderate	[23]
Immunomodulation	Meal-derived peptides, acetylated polysaccharides	Cell experiments	Weak	[52,53]
Antiviral/antiproliferative activity	Tiger nut milk, fermented plant-based beverage	Cell experiments	Weak	[58,59]

## Data Availability

No new data were created or analyzed in this study. Data sharing is not applicable to this article.
